# The Dermatology Fast Track as a Model for Integrated Care Pathways Between Emergency Medicine and Outpatient Specialty Services

**DOI:** 10.3390/medicina61122133

**Published:** 2025-11-29

**Authors:** Edoardo Cammarata, Chiara Airoldi, Elisa Zavattaro, Francesco Gavelli, Ugo Fazzini, Mattia Bellan, Paola Savoia, on behalf of the DFT Group

**Affiliations:** 1 Azienda Ospedaliero-Universitaria Maggiore della Carità di Novara, 28100 Novara, Italy; edoardo.cammarata@maggioreosp.novara.it (E.C.); ugo.fazzini@maggioreosp.novara.it (U.F.);; 2 Department of Health Science, University of Eastern Piedmont, 28100 Novara, Italy; chiara.airoldi@uniupo.it (C.A.); elisa.zavattaro@med.uniupo.it (E.Z.); 3 Department of Translational Medicine, University of Eastern Piedmont, 28100 Novara, Italy; francesco.gavelli@med.uniupo.it (F.G.); mattia.bellan@med.uniupo.it (M.B.)

**Keywords:** efficiency in healthcare, dermatological emergencies, patient flow management, healthcare integration

## Abstract

*Background and Objectives:* Dermatological conditions account for a significant proportion of Emergency Department (ED) visits but are often misclassified at triage and managed without timely specialist input. A Dermatology Fast Track (DFT) pathway was implemented to improve diagnostic accuracy, optimize resource use, and enhance integration between ED and dermatology services. *Materials and Methods:* We conducted a retrospective study of patients referred through the DFT between April 2023 and October 2024. Demographics, triage codes, diagnoses, comorbidities, prior healthcare utilization, treatments, and follow-up were analyzed. Concordance between ED and dermatology-assigned triage codes was assessed using Cohen’s kappa, and temporal trends in referrals were explored. *Results:* Of 621 patients referred, 554 were included (mean age of 47.7 years and balanced sex distribution). Most were triaged green (75.6%) or white (23.1%), and 99.5% were discharged home. Infectious dermatoses (21.1%) and eczema (17.7%) were most frequent, with age-specific variations. Combined topical and systemic therapy was prescribed in 66.1% of cases, and 30.9% were referred for follow-up. Concordance between ED and dermatology triage codes was limited (58.7% agreement; Cohen’s kappa 0.25), with frequent down-grading of priority by dermatologists. Seasonal peaks were observed, with higher demand during summer months. *Conclusions:* The DFT pathway streamlines ED care, ensuring timely management of acute dermatological conditions and reducing overcrowding. Seasonal demand fluctuations and discrepancies in triage highlight the need for targeted staff training, structured follow-up, and resource planning. Overall, the DFT is an effective model for enhancing ED efficiency, diagnostic accuracy, and patient care; however, as outcomes were assessed only in the DFT cohort and the study was conducted in a single center using Italy’s color-coded triage system, the generalizability of these findings may be limited. Multicenter studies are needed to confirm its broader applicability.

## 1. Introduction

Emergency Departments (EDs) serve as the frontline of healthcare, providing immediate access for patients with acute or unexpected medical needs. Traditionally, patient management in the ED is organized as a sequential, physician-centered pathway: after an initial nurse-led triage, patients are evaluated by an emergency physician and, when necessary, subsequently referred to a specialist. While this model is essential for the timely management of severe, complex, or undifferentiated presentations, it has several well-recognized limitations. In particular, the stepwise structure can contribute to overcrowding, prolonged waiting times, and inefficient use of scarce human and structural resources—especially when applied to less critical or highly specific conditions that may not require full emergency physician evaluation [1,2]. To address these systemic challenges, alternative organizational models have been progressively introduced. Among these, fast-track pathways have gained increasing attention as an effective strategy to streamline patient flow and improve operational efficiency. In this model, triage remains nurse-led, but patients who meet predefined criteria—thus excluding those with potentially severe or unstable conditions—are referred directly to the appropriate specialist without an intermediate evaluation by the emergency physician. By bypassing unnecessary steps, fast-track systems have been shown to reduce crowding, decrease waiting and throughput times, and improve patient satisfaction, while ensuring that specialist expertise is delivered early in the diagnostic process [3,4,5,6].

Within this framework, dermatological emergencies represent a clinically relevant yet often underestimated subset of ED consultations. While many cases involve urgent but non-life-threatening conditions—such as acute urticaria, drug eruptions, or herpes zoster—others may correspond to severe and potentially life-threatening diseases [7,8,9,10]. The absence of immediate dermatological expertise at the point of triage can lead to diagnostic errors, unnecessary hospital admissions, delays in treatment, and inefficient allocation of resources [11,12,13].

In this context, we developed a Dermatology Fast Track (DFT) pathway at the AOU Maggiore della Carità of Novara, designed to rapidly channel patients presenting with acute skin conditions from the ED to a dedicated dermatology consultation. The primary objectives of the pathway were to: (i) improve diagnostic accuracy through early specialist assessment; (ii) optimize triage classification and resource allocation; (iii) reduce inappropriate hospital admissions and unnecessary ED burden. By focusing on both clinical outcomes and healthcare process indicators, this paper aims to assess the validity of the DFT model and to describe the characteristics of patients who access it, highlighting its potential role in enhancing efficiency, resource optimization, and quality of care in dermatological emergencies.

## 2. Materials and Methods

### 2.1. Study Design, Subjects, and Methods

This retrospective study analyzed clinical data from patients who presented to the Emergency Department (ED) of the AOU Maggiore della Carità of Novara and were subsequently referred through the Dermatology Fast Track (DFT) pathway to the Dermatology Unit of the same hospital between April 2023 and October 2024. Patients were considered eligible for referral to the DFT pathway according to predefined inclusion and exclusion criteria, as summarized in Table 1. Patients presented with lesions in anatomical regions potentially requiring multidisciplinary evaluation (e.g., periorbital, oral, genital) were excluded, as these cases are more effectively managed in the emergency department with access to multiple specialists.

Patients included in this study were those with complete clinical documentation available for both the ED assessment and the dermatology consultation. Patients with incomplete or inconsistent records, as well as those who failed to attend the scheduled dermatology consultation, were excluded from the analysis.

### 2.2. Data Collection

For each patient included in the study a comprehensive set of clinical, demographic, and process-related variables was systematically collected. Demographic characteristics encompassed age and sex Clinical urgency at presentation was characterized using the triage color code assigned upon ED admission and subsequently updated at discharge, in accordance with the standardized Italian emergency classification system. Diagnostic data included the initial diagnosis formulated by the emergency physician and the final diagnosis established during dermatology consultation, allowing evaluation of diagnostic concordance and the frequency of revised or corrected diagnoses.

Additionally, the presence of comorbidities was recorded, based either on documented medical history within the clinical record or on patient-reported information obtained during the encounter. Data were also gathered regarding previous healthcare utilization related to the presenting skin condition, including prior ED visits for the same complaint or previous scheduled dermatology consultations, in order to assess patterns of recurrence and continuity of care. Therapeutic management was characterized by documenting the type of treatment prescribed during the dermatology consultation, categorized as topical therapy, systemic therapy, or a combination of both. Information on follow-up strategies was also collected, including whether a dermatology follow-up appointment was scheduled and its intended timing. Finally, to evaluate post-consultation outcomes and potential persistence or progression of symptoms, any subsequent ED visits occurring after the dermatology assessment were recorded.

### 2.3. Data Management and Statistical Analysis

Data were extracted from electronic hospital databases, anonymized, and organized into a dedicated Excel file. Categorical variables were summarized as absolute frequencies and percentages, whereas continuous variables were reported as mean ± standard deviation (SD) or as median with interquartile range (IQR), depending on data distribution. Concordance between the triage color code assigned at ED admission and that revised by the dermatologist at discharge was assessed using agreement percentages and Cohen’s kappa coefficients (both simple and weighted), with corresponding 95% confidence intervals (95% CI). The weighted kappa coefficient was used to assess the degree of agreement for an ordinal variable, taking into account the magnitude of disagreements between categories. Weights were calculated using the Cicchetti-Allison method, which penalizes small disagreements less than larger ones. Interpretation of kappa values followed the Landis and Koch scale (<0 no agreement, 0−0.20 slight agreement, 0.21−0.40 fair agreement, 0.41−0.60 moderate agreement, 0.61−0.80 substantial agreement, 0.81−1 almost perfect agreement). Comparison between Dermatological Diagnosis and categorical age was also performed using chi-square test. Temporal trends in monthly ED visits were explored through time-series graphical representation.

All statistical analyses were performed using SAS software (version 9.4, SAS Institute Inc., Cary, NC, USA) and R software (R Foundation for Statistical Computing, Vienna, Austria). Statistically significant threshold was set to 0.05, two tailed. 

## 3. Results

**Figure 1:**
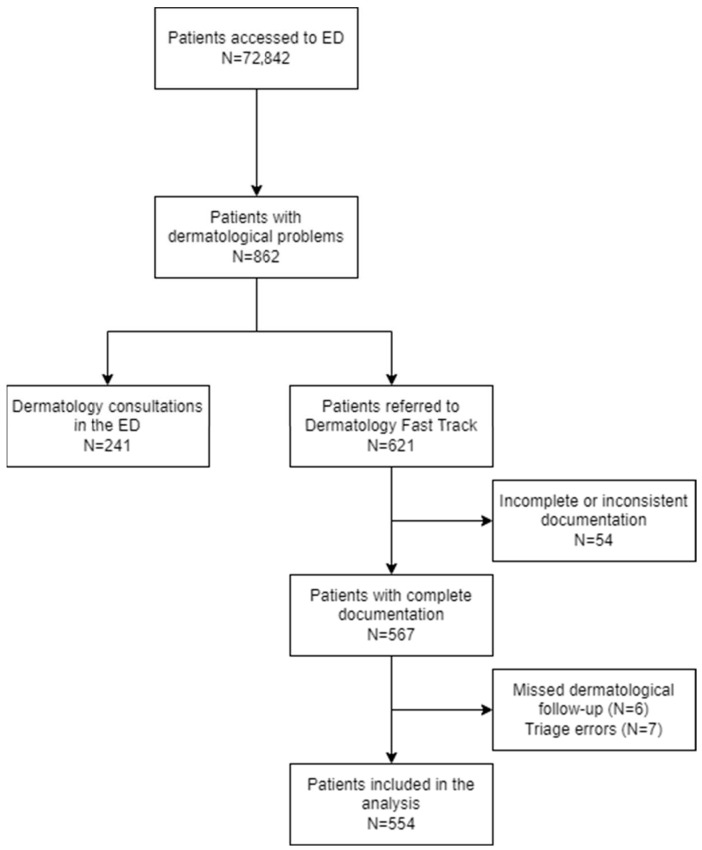
Participant flow chart for the study population. ED: Emergency Departement.

During the 17-month follow-up period, a total of 72,842 emergency department (ED) visits were recorded. Of these, 862 cases (1.18% of the total) required a dermatological specialist consultation. Among them, 241 patients (28.0%) were first evaluated by ED physicians and subsequently referred for dermatological consultation, while 621 patients (72.0%) were directly sent to the dermatology fast track based on predefined criteria (Figure 1).

Of these 621 patients, 54 were excluded due to incomplete demographic or clinical data. Among the remaining 567 patients, an additional 13 were excluded: 7 patients (1.23%) due to triage errors, as they did not present with dermatological complaints (2 white codes and 5 green codes), and 6 patients (1.06%) who left the ED before undergoing the dermatological consultation (3 white codes and 3 green codes).

The final study cohort comprised 554 patients for whom complete data were available, including triage color code and diagnosis at both ED admission and dermatology evaluation. This population was therefore used for all subsequent statistical analyses.

### 3.1. Sample Characteristics

A total of 554 patients were considered, with a balanced sex distribution (51.6% female, 48.4% male) and a mean age of 47.7 years (SD ± 20.1; median 47.3). The most frequent comorbidities were metabolic disorders (12.1%), cardiovascular diseases (4.2%), and pulmonary diseases (1.3%). Most patients were triaged green (75.6%), followed by white (23.1%), while only 1.3% received a blue code. Nearly all (99.5%) were discharged home.

Regarding prior healthcare utilization, 71.7% had no previous visits, 6.9% had urgent visits only, 12.4% scheduled visits only, and 9.0% both.

The most common dermatological diagnoses were cutaneous infections (21.1%) and eczema (17.7%), followed by entomodermatoses (12.5%), burns (6.1%), cystic lesions and scabies (both 5.8%), urticaria (4.7%), tick bites (3.3%), and drug eruptions (2.7%). Less frequent diagnoses included psoriasis, pruritus with associated lesions, inflammatory skin lesions, cutaneous neoplasms, and non-eczematous erythematous lesions (Table 2).

Treatment was prescribed in almost all cases: 66.1% received combined topical and systemic therapy, 22.2% topical only, and 8.1% systemic only; 3.6% received no treatment. The 30.9% of patients were referred for follow-up specialist visits.

Table 3 shows the distribution of diagnoses by age group. Most patients were 30–65 years (55.8%), followed by <30 years (23.8%) and >65 years (20.4%). Cutaneous Infections and Infestations predominated in younger patients (<30 years), cutaneous neoplasms in older patients (>65 years), and burns were most frequent among adults aged 30–65 years. However, differences between diagnosis and age were not statistically significant (*p* = 0.1247).

### 3.2. Concordance Between Admission and Discharge Priority Codes

Table 4 illustrates the relationship between the triage priority code assigned at admission and the code defined by the dermatology specialist at discharge.

The main diagonal shows cases of perfect agreement between codes, totaling 325 patients out of 554, corresponding to an overall accuracy of 58.66%. Concordant cases include 106 white/white, 212 green/green, and 7 blue/blue codes. Elements below the main diagonal indicate overestimation of priority at triage. Specifically, 207 patients (37.36%) initially assigned green were downgraded to white by the specialist. Elements above the main diagonal represent cases where triage underestimated clinical severity. This occurred in 22 patients (3.97%), who received a higher priority code at discharge compared to admission.

The degree of statistical agreement, measured using Cohen’s kappa, was very low. The unweighted kappa was 0.248 (95% CI: 0.186–0.311), and the weighted kappa was 0.262 (95% CI: 0.197–0.327), indicating limited concordance between emergency triage and dermatology assessment.

### 3.3. Temporal Trend of Visits

**Figure 2:**
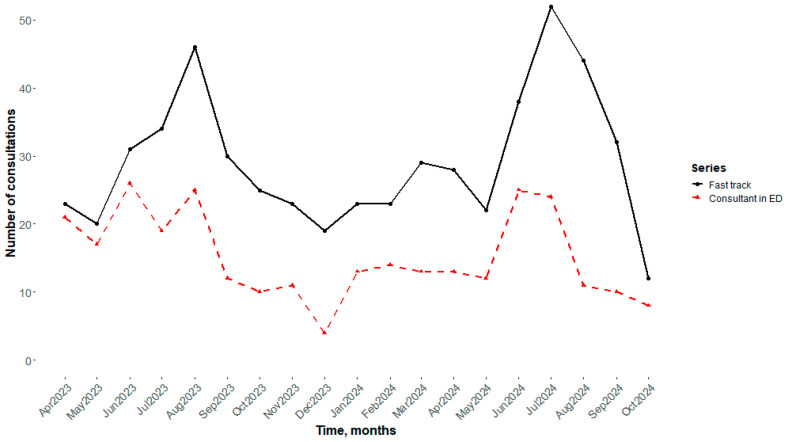
Temporal trend of visits. Black line indicates the Fast track consultations, while the red line the consultations performed directly in Emergency Departments (ED).

Analysis of the monthly distribution of visits to the dermatology fast-track service (black line) and consultant in ED (red line) over the study period suggests a clear seasonal pattern (Figure 2). The number of consultations tended to increase during the summer months, with peaks observed in August 2023 and July 2024. Lower numbers of visits were recorded during the winter months, particularly in December 2023 and October 2024. This pattern indicates a higher demand for dermatological consultations in the warmer months.

## 4. Discussion

Overcrowding and limited resources remain persistent and critical challenges for Emergency Departments (ED), underscoring the need for of innovative organizational strategies capable of optimizing patient flow and improving overall efficiency. Among these strategies, the Fast Track was developed with the specific aim of alleviating ED congestionby directing selected patients with acute conditions straight to the specialist evaluation. In this study, we provide a comprehensive analysis of the demographic, clinical, and management characteristics of patients referred to the Dermatology Fast Track (DFT) pathway at the Emergency Department of the AOU Maggiore della Carità of Novara over an 18-month period. By examining patterns of access, diagnostic concordance, triage accuracy, therapeutic decisions, and follow-up strategies, our analysis provides valuable insight into how the DFT functions in real-world clinical practice. Our findings confirm the clinical and organizational advantages of the DFT in delivering timely and appropriate care for acute dermatological conditions, while also identifying areas where further refinement of the pathway could enhance its effectiveness. At the same time, the results highlight specific areas where targeted refinements—such as expanded triage criteria, enhanced communication between ED and dermatology teams, or improved patient education—could further strengthen the pathway’s performance. Overall, this study contributes to the growing evidence supporting fast-track approaches as a means to improve quality of care, optimize resource utilization, and enhance operational efficiency in Emergency Medicine.

The study population was heterogeneous in terms of both age and sex, with a nearly equal distribution between males and females and a mean age of 48 years. These findings underscore the transversal nature of dermatological diseases, which can affect individuals at any stage of life [14,15,16]. Infections emerged as the most frequent reason for ED access, accounting for over 20% of all diagnoses, consistent with existing literature [3,4,5,17,18]. Eczema and entomodermatoses were also common, while less frequent but clinically significant conditions such as urticaria, drug eruptions, psoriasis, and cutaneous neoplasms were also observed. Age-specific differences were evident: scabies, entomodermatoses, and tick bites predominated in younger patients, whereas neoplasms were more frequent in those over 65 years, in line with the cumulative effect of oncogenic risk factors.

The vast majority of patients were discharged home after dermatology consultation, with only rare cases requiring hospitalization. This outcome, also supported by the patient characteristics and the low number of associated comorbidities, mirrors findings from other European cohorts [19,20], underscoring the DFT’s ability to decompress general ED activity by avoiding inappropriate admissions. Treatment was prescribed in almost all cases, with a predominance of combined topical and systemic therapy. This reflects the acute or complex nature of many dermatological emergencies but also highlights the need for careful therapeutic appropriateness to prevent over- or undertreatment. Approximately one-third of patients were referred for follow-up, confirming that the DFT not only serves as a first assessment tool but also plays an important role in continuity of care.

Another relevant observation concerned prior healthcare utilization. While most patients (≈72%) had no history of previous visits, nearly one-third had either scheduled or urgent prior contacts, and approximately 9% had both. Notably, a significant proportion of patients frequently visit the ED for non-urgent conditions, often due to limited access to primary care or the need for routine prescriptions and minor symptom management, thereby contributing to ED overcrowding [21,22]. These findings suggest that the DFT also plays an important monitoring role for patients already known to the healthcare system, some of whom may benefit from structured follow-up programs that integrate outpatient care with fast-track pathways.

A critical issue that emerged was the limited concordance between ED triage codes and those reassessed by dermatologists at discharge. Overall agreement was poor, with a substantial proportion of cases downgraded from green to white, reflecting a systematic overestimation of urgency at admission. This observation aligns with previous reports in the literature [23], which found that nearly a quarter of dermatologic patients are assigned an inappropriately high level of urgency. Importantly, only a small minority of cases were underestimated, and no serious cases were missed, confirming the reliability of our patient selection criteria. It should be noted that under triage entails the risk of delays in diagnostic and therapeutic interventions, which may be potentially harmful for certain categories of high-risk patients [24]. Overall, our results highlight the challenges non-specialists face in accurately assessing dermatological urgency and underscore the added value of early specialist input. Targeted training programs for triage staff, with a focus on dermatology, could further improve triage accuracy and reduce misclassification without compromising patient safety.

The analysis of temporal distribution for patient admissions via the DFT pattern revealed a clear seasonal pattern, with peaks during the summer months. This trend can be attributed to environmental and behavioral factors, such as increased incidence of sunburns, insect bites, and maceration-related infections, combined with reduced availability of general practitioners during holiday periods. Other studies have documented similar seasonal fluctuations in dermatological visits [25,26]. For instance, a study analyzing emergency department visits due to sunburn found that the prevalence and cost of such visits increased during the summer months, highlighting the seasonal nature of these conditions [27]. Also, bacterial skin infections were more frequently reported during the summer months [28] and an increased occurrence of insect bites during summer has been documented [29]; both circumstances may have contributed to a rise in dermatological complaints during this period. These findings suggest the need for resource planning that accounts for predictable demand surges, ensuring adequate staffing and preparedness to manage seasonal increases in dermatological emergencies.

Overall, this study confirms that the Dermatological Fast Track (DFT) is an effective organizational strategy to streamline Emergency Department workflows, delivering timely and appropriate care for acute dermatological conditions while alleviating ED overcrowding. The heterogeneity of the patient population, seasonal fluctuations in case distribution, and inconsistencies in triage coding highlight both the strengths of the pathway and opportunities for further optimization. Targeted training for triage staff, structured integration with outpatient follow-up programs, and resource planning adapted to predictable seasonal surges may enhance the efficiency and safety of the DFT model. 

Nonetheless, several limitations should be acknowledged. First, outcomes are reported exclusively for the DFT cohort, without a comparison group receiving standard ED care. This limits the generalizability of our findings and precludes definitive conclusions regarding the relative effectiveness of the DFT pathway compared to usual emergency management. Second, the study was conducted in a single center in Italy, where the color-coded triage system is applied. Because this triage system is not universally adopted, the replicability and applicability of our findings to settings with different triage procedures or organizational structures may be limited. Moreover, the retrospective design and reliance on existing clinical records may have introduced information bias, and some patient characteristics or outcomes could not be captured systematically.

Despite these limitations, our analysis provides a detailed overview of patient demographics, clinical presentations, and management patterns within a real-world DFT pathway, highlighting both its potential benefits and areas for improvement. Future multicenter prospective studies—including control groups, economic analyses, and patient-reported outcomes—are warranted to validate the DFT model, assess its broader applicability, and guide its adaptation in healthcare systems with different triage methods or resource constraints.

## 5. Conclusions

The Dermatology Fast Track appears to be an effective organizational model for the rapid and appropriate management of acute skin conditions in the Emergency Departments (ED), as suggested by improvements in diagnostic precision, resource use, and continuity of care within our cohort. However, since this study was conducted in a single center without a comparative arm and did not evaluate outcomes such as waiting time reduction or economic impact, the generalizability of these findings is limited. With targeted refinements—particularly in triage training, seasonal planning, and structured follow-up—the DFT has the potential to serve as a promising model for integrated emergency-specialist care, pending validation through multicenter and comparative studies.

## Figures and Tables

**Table 1:** Inclusion and exclusion criteria for Dermatology Fast Track (DFT) pathway.

Inclusion Criteria	Exclusion Criteria
Localized skin lesions (<10% BSA), excluding periorbital and oral regions *; erythematous, edematous, vesicular, urticarial, or crusted	Altered vital signs
Arthropod or parasite bites (e.g., tick bite), with or without parasite presence	Diffuse erythematous, urticarial, or bullous skin lesions and/or lesions associated with systemic symptoms
First- or second-degree burns <10% BSA (excluding face, neck, genitals, groin, axilla *, burns with dangerous mechanism or inhalation)	Chemical skin contamination
Single pre-existing skin lesion with recent changes and/or bleeding (e.g., melanocytic lesion)	Lesions suspicious for physical abuse or maltreatment
Dehiscence of surgical wounds (after dermatological procedures)	Requests for medico-legal investigations by judicial authorities
Nonspecific or post-traumatic abrasions (in trauma already evaluated)	Patients referred directly from other healthcare facilities (e.g., GPs, out-of-hours services, nursing homes)
Genital ulcers	

* Lesions requiring multidisciplinary evaluation (e.g., periorbital, oral, genital) were excluded.

**Table 2:** Main dermatological diagnosis for patients referred to the DFT pathway.

Dermatological Diagnosis		*N* (%)	
Cutaneous Infections and Infestations	Infectious ^1^	117 (21.12%)	236 (42.60%)
	Entomodermatoses	69 (12.45%)	
	Scabies	32 (5.78%)	
	Tick bites	18 (3.25%)	
Inflammatory Skin Diseases	Eczema	98 (17.69%)	185 (33.39%)
	Urticaria	26 (4.69%)	
	Drug eruptions	15 (2.71%)	
	Psoriasis	14 (2.53%)	
	Pruritus and associated lesions	12 (2.17%)	
	Inflammatory skin lesions	11 (1.99%)	
	Non-eczematous erythematous lesions	9 (1.62%)	
Burns		34 (6.14%)	34 (6.14%)
Skin Tumors and Cystic Lesions	Cystic lesions	32 (5.78%)	42 (7.58%)
	Neoplasms	10 (1.81%)	
Other		57 (10.29%)	57 (10.29%)

^1^ Among these, 63 patients (53.8%) presented with viral infections, 46 (39.3%) with bacterial infections, and 8 (6.8%) with fungal infections.

**Table 3:** Distribution of major dermatological diagnoses by age group (<30 years, 30–65 years, >65 years).

Diagnosis	<30 Years *N* (%)	30–65 Years *N* (%)	>65 Years *N* (%)
Cutaneous Infections and Infestations	63 (47.73)	126 (40.78)	47 (41.59)
Inflammatory Skin Diseases	44 (33.33)	102 (33.01)	39 (34.51)
Burns	7 (5.30)	26 (8.41)	1 (0.88)
Skin Tumors and Cystic Lesions	6 (4.55)	25 (8.09)	11 (9.73)
Other	12 (9.09)	30 (9.71)	15 (13.27)
Total	132 (23.83%)	309 (55.78%)	113 (20.40%)

Values are presented as *N* and %, calculated with respect to the column total.

**Table 4:** Concordance between admission and discharge triage codes.

Admission Code *	Discharge Code White	Discharge Code Green	Discharge Code Blue	Total
White	106	22	0	128
Green	207	212	0	419
Blue	0	0	7	7
Total	313	234	7	554

* Triage codes follow the Italian emergency system: green = low priority, white = non-urgent, blue = special cases.

## Data Availability

Data are unavailable due to privacy and ethical restrictions.

## Abbreviations

The following abbreviations are used in this manuscript:

DFT	Dermatologic Fast Track
ED	Emergency Department
